# Una nueva perspectiva en el cribado del cáncer de próstata

**DOI:** 10.1515/almed-2023-0034

**Published:** 2023-07-21

**Authors:** Xavier Filella, Álvaro González, Josep Maria Augé, Antonio Barco, Rosa Carbonell, María Jesús Gaspar, Antonio Martínez-Peinado, Clara Pérez Barrios, Marta Sánchez-Carbayo, José Diego Santotoribio, Jaume Trapé

**Affiliations:** Servicio de Bioquímica y Genética Molecular (CDB), Hospital Clínic, IDIBAPS, Barcelona, España; Servicio of Bioquímica, Clínica Universidad de Navarra, Pamplona, España; Servicio de Bioquímica Clínica, Hospital Universitario Virgen Macarena, Sevilla, España; Laboratorio de Bioquímica, Hospital Universitario San Juan, San Juan, Alicante, España; Servicio de Análisis Clínicos, Hospital Universitario Príncipe de Asturias, Alcalá de Henares, Madrid, España; Unidad Clínica de Gestión de Análisis Clínicos, Sección de Genética Molecular, Hospital Universitario Reina Sofía, Córdoba, España; Laboratoro, Hospital Universitario Puerta del Hierro, Majadahonda, Madrid, España; Departamento de Ciencias de la Salud, Universidad Pontificia, Salamanca, España; Laboratorio, Hospital Universitario Virgen del Rocio, Sevilla, España; Laboratorio, Althaia Xarxa Assitencial Universitària, Manresa, España

**Keywords:** cribado, antígeno específico de la próstata (PSA), cáncer de próstata

## Abstract

El cribado del cáncer de próstata mediante la medida del antígeno específico de la próstata (PSA) ha sido objeto de una intensa polémica. Los beneficios derivados en cuanto a reducción en mortalidad del cribado organizado del cáncer de próstata se han acompañado de un importante sobrediagnóstico, que se ha traducido en tratamientos innecesarios y numerosos efectos adversos. Por ello, las recomendaciones de las sociedades científicas han sido cada vez más restrictivas. En los últimos años se han hecho diversas propuestas para reconsiderar el enfoque sobre el cribado del cáncer de próstata, con objeto de superar la oposición entre cribar a todo el mundo o no cribar a nadie y a reconsiderar el PSA como una herramienta que permita un balance favorable entre beneficios y riesgos. En este contexto, hay que destacar la propuesta que la European Association of Urology dirigía a la Comisión Europea replanteando el cribado del cáncer de próstata en función de la medida de un PSA basal. Recientemente, la Comisión Europea ha recomendado la implementación de programas organizados para el cribado del cáncer de próstata para hombres de hasta 70 años basado en medidas de PSA en combinación con la resonancia magnética como prueba de seguimiento.

## Introducción

El cáncer de próstata (CaP) es en España el tumor más frecuente en varones, contabilizándose 30.884 nuevos diagnósticos en 2022, y si bien la supervivencia neta de este tumor a los 5 años es del 89,8 %, fue responsable de 5922 muertes en 2020 [1]. La disponibilidad de tratamientos curativos cuando se aplican en pacientes con la enfermedad en un estadio inicial hace que el diagnóstico precoz sea una oportunidad para mejorar su pronóstico.

Durante muchos años el cribado mediante la medida del antígeno específico de la próstata (PSA) ha sido objeto de múltiples polémicas, pese a que los resultados obtenidos por el European Randomized Study of Screening for Prostate Cancer (ERSPC) muestran una disminución de la mortalidad debida al CaP en el grupo de cribado frente al grupo control del 21 % tras 13 años de seguimiento [2]. Además, en la actualización de datos publicada con 16 años de seguimiento [3] se constata que en el grupo de varones que atendieron a una ronda de cribado la reducción en la mortalidad por CaP fue del 25 %, mientras que dicha reducción fue del 48 % en el grupo que hizo varias rondas de cribado. De la misma manera, en esta actualización se indica que el número de hombres que deben ser cribados para prevenir una muerte disminuyó de 742 a 570 cuando el tiempo de seguimiento aumentó de 13 a 16 años.

De hecho, los resultados del ERSPC contrastan con los resultados negativos reportados por parte del ensayo americano PLCO (siglas correspondientes al Prostate, lung, colorectal, and ovarian screening trial) que en sus distintas actualizaciones, cada vez con mayor tiempo de seguimiento hasta alcanzar un tiempo mediano cercano a los 17 años, no se observa ninguna diferencia entre el grupo de cribado y grupo control [4, 5]. Tampoco se observaron diferencias en un estudio realizado en el Reino Unido que incluyó 419.582 hombres de entre 50 y 80 años [6].

En todo caso deben subrayarse dos particularidades de estos dos ensayos. Por un lado, el ensayo norteamericano tiene una elevada tasa de contaminación con medidas de PSA en la rama control, que ha sido estimada entre el 42 y el 50 %, por lo que no ofrece una respuesta definitiva sobre la utilidad del cribado, como han reconocido los mismos autores del ensayo [7]. Por otro lado, el estudio británico se basa en una determinación puntual de PSA y no en varias medidas a lo largo de los años como sucede con los ensayos ERSPC y PLCO, y además tiene un tiempo mediano de seguimiento de tan solo 10 años y una adherencia al protocolo de solo el 40 %, hechos que como subrayan los autores del estudio pueden dificultar la valoración de la utilidad del cribado con PSA del CaP.

En todo caso, sin embargo, los beneficios derivados del cribado con PSA han supuesto un importante sobrediagnóstico de tumores indolentes que se ha traducido en tratamientos innecesarios pero que se acompañan de perjuicios evidentes para el paciente y que abarcan desde la incontinencia urinaria a la impotencia.

## Pros y contras de la restricción del PSA como herramienta de cribado del cáncer de próstata

En 2012, el US Preventive Services Task Force (USPSTF), como consecuencia de los datos publicados por el estudio PLCO tras 13 años de seguimiento [4], se posicionaba, rotundamente en contra del cribado puesto que consideró que el balance entre beneficios -que estimaba escasos- y riesgos era negativo [8]. Las recomendaciones sobre el uso de PSA en el cribado del CaP de la mayoría de las sociedades científicas han sido a partir de aquel momento mucho más restrictivas, de manera que el número de determinaciones de PSA empezó a disminuir. De hecho, la no realización de medidas de PSA en el cribado del CaP era una de las cinco recomendaciones incluidas en la iniciativa Choosing Wiselys que el American Board of Internal Medicine Foundation impulsaba, con gran repercusión, en 2016 [9].

En este sentido, se expresa también el documento “Decisiones inteligentes desde el laboratorio: de elegir sabiamente a no hacer” de AEBM-ML, que en 2021 actualiza el anterior texto de 2015, al señalar que no se ha conseguido demostrar de manera fehaciente que el cribado con PSA reduzca la mortalidad, recomendando que la decisión debe tomarla el paciente tras ser convenientemente informado de los beneficios y riesgos que conlleva [10]. No muy distinta es la posición de la Sociedad Española de Medicina de Familia y Comunitaria al recomendar en 2017 “no realizar de forma sistemática la determinación de PSA a individuos asintomáticos sin antecedentes familiares de primer grado de cáncer de próstata” [11]. También es sumamente restrictiva la postura del Ministerio de Sanidad, Servicios Sociales e Igualdad que en la ponencia de Cribado Poblacional de la Comisión de Salud Pública, fechada en abril de 2019, subraya que el balance entre riesgos y beneficios del cribado del CaP con PSA es negativo [12].

Las recomendaciones contrarias al cribado han causado un retraso en el diagnóstico de la enfermedad. Así, Bandini et al. [13] comunican un incremento en la incidencia de nuevos casos diagnosticados con metástasis tras analizar los datos de la base SEER (Surveillance, Epidemiology and End Results). Los autores observan que, entre 2004 y 2014, la edad de presentación de CaP diseminado disminuyó, mientras que el número de pacientes que debutó con metástasis aumentó del 1,9 a 2,4 por 100 000 habitantes.

## El PSA basal como una herramienta útil en la selección de pacientes

En el curso de los últimos años diversos autores han hecho propuestas para reconsiderar el enfoque sobre el cribado del CaP basado en determinaciones de PSA. Diversas propuestas -descritas como cribado inteligente, estrategia de cribado personalizada o cribado individualizado- se encaminan a superar la oposición entre cribar a todo el mundo o no cribar a nadie y a reconsiderar la utilización del PSA como una herramienta que permita un balance favorable entre riesgos y beneficios [14].

Se ha señalado en repetidas ocasiones la falta de especificidad del PSA en pacientes con enfermedades benignas de la próstata, a menudo en relación a un aumento del volumen prostático. Sin embargo, su especificidad es mucho mayor en sujetos jóvenes, cuando la próstata no ha empezado a agrandarse. Este es el dato que explica la capacidad del PSA para predecir el ulterior diagnóstico de un CaP, tal como mostraban los resultados publicados en 1994 por Stenmann et al. [15] al señalar que una elevación de PSA superior a 2,5 μg/L permitía predecir el diagnóstico de un CaP a lo largo de la década siguiente a la medición del PSA.

Estudios posteriores han documentado con mayor amplitud esta observación, si bien únicamente un estudio reciente ha ofrecido datos en base a la evaluación de una amplia población. Se trata de un análisis secundario de los datos del ensayo americano PLCO que incluye 10.968 individuos de edades comprendidas entre los 55 y los 60 años y que cuenta con un tiempo de seguimiento de 13 años. Los autores observan una relación entre el posterior diagnóstico de un CaP clínicamente significativo y la concentración del PSA basal y concluyen que la frecuencia del cribado puede espaciarse en individuos con un PSA basal menor a 2 μg/L y que probablemente se podría discontinuar cuando el PSA es menor a 1 μg/L [16].

La implementación de una estrategia personalizada de cribado del CaP en función de un PSA basal permitiría, según las evidencias disponibles, mejorar los resultados del cribado, reduciendo el sobrediagnóstico de la enfermedad al disminuir el número de determinaciones de PSA en pacientes de cierta edad, en los cuales la probabilidad de un falso positivo relacionado con patología benigna de la próstata es más frecuente. En segundo lugar, supondría un notable ahorro en cuanto a determinaciones de PSA, puesto que en muchos pacientes con una concentración basal de PSA baja se discontinuaría, en ausencia de otros datos que lo aconsejasen, la realización de determinaciones de PSA.

## Nuevas herramientas en la detección del cáncer de próstata

El cambio de perspectiva en el cribado del CaP que se ha producido en el curso de los últimos años es, sin embargo, aun más amplio. Por un lado, la vigilancia activa ha sido propuesta como una actitud válida para pacientes con un CaP con una baja probabilidad de progresión, en los cuales puede diferirse el tratamiento hasta el momento en que se pueda evidenciar, en función del cambio en las características del tumor, que es realmente imprescindible [17]. Por otro lado, la resonancia magnética multiparamétrica (RMmp) permite obtener información sobre el riesgo de tener un CaP y en definitiva decidir en última instancia la realización de una biopsia. En este sentido hay que destacar, como subraya el estudio Göteborg 2 [18], la capacidad de la RMmp para detectar tumores clínicamente significativos, disminuyendo el sobrediagnóstico de la enfermedad. Finalmente, disponemos de evidencias que remarcan el interés de nuevos biomarcadores, como 4kscore (algoritmo que combina PSA total, libre e intacto y la calicreína 2 junto a información referente a la edad del paciente, el resultado del tacto rectal y la posible existencia de una biopsia negativa previa), Prostate Health Index (PHI, que combina el PSA total y libre y el [-2]proPSA), o el test Estocolmo 3 (S3M, que combina cinco proteínas, PSA total y libre, calicreína 2, MSMB y MIC1, junto a un panel de 254 SNPs relacionados con CaP y datos demográficos y clínicos, que incluyen historia familiar y biopsias prostáticas previas), que ofrecen una mayor especificidad que el PSA y, además, se relacionan con la agresividad del tumor, permitiendo una reducción del sobrediagnóstico [19].

A este respecto destaca el propio objetivo del test 4kscore, encaminado a detectar no ya CaP, sino únicamente CaP de alto grado (grado de Gleason 7 o superior). Los resultados reportados en un estudio multicéntrico norteamericano que involucra 1,012 varones indican que permite reducir el número de biopsias en un 30 %, retrasando el diagnóstico en solo el 1,3 % de los pacientes, siendo el área bajo la curva obtenida de 0,821 [20]. Asimismo, las evidencias disponibles sugieren que un resultado de 4kscore basal permite estratificar los pacientes en relación al riesgo de desarrollar un CaP metastásico a largo plazo, incluso con mayor efectividad que el PSA [21].

Por su lado, PHI se relaciona con la agresividad del tumor, tal y como han mostrado Maxeiner et al. [22] al observar que el valor de este índice se relaciona con la probabilidad de desarrollar una recidiva bioquímica en una serie de 437 pacientes tratados con prostatectomía radical. Igualmente, la especificidad de PHI es superior a la de PSA o a la del porcentaje de PSA libre tanto en la detección de CaP como en la de CaP con un riesgo intermedio o alto de progresión, particularmente en pacientes con un tamaño de la próstata pequeño (con un área bajo la curva para PHI de 0,817) o mediano (con área bajo la curva para PHI de 0,759) [23]. Los pacientes más jóvenes, con una próstata de menor volumen, serían por tanto los que más pueden beneficiarse de la medición de PHI. No menos interesantes son los resultados reportados por Druskin et al. [24] que muestran que el área bajo la curva aumenta de de 0,83 a 0,90 cuando la densidad de PHI, calculado como la relación entre PHI y el volumen prostático en mL, se añade a un modelo base que incorpora la edad, la existencia de una biopsia negativa previa y el resultado de RMmp.

Los resultados comunicados para el test S3M también muestran una elevada especificidad y su relación con la agresividad del tumor. A este respecto, destaca el estudio de cribado desarrollado en Stavanger (Noruega) [25], en que se observa que la sustitución de la medida de PSA por el test S3M aumenta la detección de pacientes con un CaP con un grado de Gleason 7 o superior, a la vez que disminuye el número de tumores clínicamente no significativos que se diagnostican.

## El debate iniciado con la propuesta de la European Association of Urology

El objetivo de un programa de cribado del CaP debería estar encaminado a disminuir el número de biopsias innecesarias y el sobrediagnóstico de la enfermedad, así como a aumentar el diagnóstico de tumores clínicamente significativos para disminuir la mortalidad relacionada con el cáncer y el número de pacientes que debutan con metástasis.

Por otro lado, la conmutabilidad de resultados entre los distintos ensayos disponibles para medir PSA permitiría reducir las actuales ineficiencias y aseguraría la calidad del programa, evitando errores en la selección de pacientes para biopsia cuando se emplea un nivel de corte universal para ensayos que miden concentraciones de PSA distintas. La introducción por parte de la OMS en 1999 de un estándar internacional ayudó a reducir la variabilidad entre ensayos, si bien, pese a todo, en la actualidad, siguen habiendo diferencias en la concentración de PSA medida con ensayos distintos [26], [27], [28] que no siempre han sido convenientemente reflejadas en las guías clínicas [29]. En nuestra opinión, por tanto, son necesarios nuevos esfuerzos por parte de los fabricantes con objeto de estandarizar al máximo los ensayos para medir PSA, de manera que se disponga de límites de decisión universalmente aceptables.

En este contexto, hay que destacar la propuesta impulsada a partir de 2017 desde la European Association of Urology (EAU) con objeto de reconsiderar el enfoque del cribado del CaP en función de la medida de un PSA basal [30], [31], [32]. Por su lado, recientemente, la Comisión Europea ha hecho pública una iniciativa para intensificar el cribado del cáncer en base a los datos actualmente disponibles con el objetivo de detectar cánceres en una etapa inicial y, en definitiva, mejorar el pronóstico de la enfermedad. Así, en un documento con fecha del 20 de septiembre de 2022, recomienda a los Estados miembros la implementación de programas organizados para el cribado del CaP para hombres de hasta 70 años sobre la base de medidas de PSA, en combinación con la resonancia magnética como prueba de seguimiento [33, 34]. Para ello se deberían tener en cuenta las particularidades de cada Estado miembro, tanto en referencia a la tecnología disponible como a la incidencia del CaP en cada territorio. El programa EU4-Health ha aprobado recientemente el consorcio Prostate cancer Awareness and Initiative Screening in the European Union (Praise-U) [35]. Se trata de un proyecto de tres años que reúne un equipo multidisciplinar compuesto por investigadores de 25 instituciones pertenecientes a 12 países miembros y que tiene como objetivo reducir la morbilidad y la mortalidad del CaP, evitando al mismo tiempo el sobrediagnóstico, a través del diseño de un algoritmo adaptado a cada país para el cribado de este tumor.

## Conclusiónes

Los resultados disponibles sugieren que la medición de un PSA basal puede ser útil para discriminar el grupo de pacientes que pueden beneficiarse de ser incluidos en un programa de cribado, evitando los posibles perjuicios para el resto de pacientes. Indudablemente, la introducción de la RMmp en el diagnóstico del CaP ha significado una notable mejora que disminuye el sobrediagnóstico y aumenta la eficacia de los programas de cribado. Finalmente, creemos son necesarios nuevos estudios prospectivos y multicéntricos que confirmen los datos disponibles respecto a los nuevos biomarcadores tanto en referencia a tener mayor especificidad que el PSA como a su posible capacidad, como ha sido publicado respecto a 4kscore, para seleccionar el grupo de pacientes con un riesgo elevado de desarrollar un CaP agresivo. Creemos, en definitiva, que, a la vista de los datos actualmente disponibles y siguiendo las directrices de la Comisión Europea, se hace necesario abrir un período de reflexión con objeto de replantear las actuales recomendaciones de las sociedades científicas y las disposiciones de las autoridades sanitarias acerca del cribado del CaP y sobre el empleo del PSA en su detección.
